# Nicotine Patch Prescription Fulfillment Rates for Emergency Department Patients

**DOI:** 10.5811/westjem.2021.2.49932

**Published:** 2021-05-14

**Authors:** Charles L. Emerman, David Tracy, Jonathan Siff

**Affiliations:** Case Western Reserve University, Department of Emergency Medicine, Cleveland, Ohio

## Abstract

**Introduction:**

Smoking cessation has significant health benefits, and the emergency department (ED) can be an important venue for smoking cessation counseling. Nicotine replacement therapy with transdermal patches has been shown to be associated with smoking cessation in a variety of studies. This study evaluated fulfillment rates for prescriptions for nicotine replacement transdermal patches (NRT-P) from the ED.

**Methods:**

We conducted a retrospective review of all patients receiving a prescription for a NRT-P product from January 2018–October 2019. Charts were reviewed to gather data including age, gender, presence of chronic heart or lung problems, and health insurance. We assessed the fulfillment rate of prescriptions using the Surescripts system, which is a functionality within our electronic health record system that queries participating pharmacies. Statistical analysis was conducted to determine associations between fill rates and the other variables collected from charts.

**Results:**

We had follow-up on 500 patients prescribed nicotine patches. Of those patients, 44% filled their prescriptions. Those who filled their prescriptions were more likely to be female and have a history of chronic lung disease. Self-pay patients were least likely to fill their prescriptions. Overall, we had evidence of smoking cessation in 13% of patients.

**Conclusion:**

This study found that a substantial proportion of patients fail to fill their NRT-P prescriptions. Further work on means of enhancing fulfillment rates is warranted.

## INTRODUCTION

Smoking continues to constitute a major health issue with estimates that around 7% of emergency department (ED) visits are related to tobacco use.^1^ Emergency physicians are in a position to help patients with smoking cessation. In a study of ED patients, about 36% of them were in the preparation stage, indicating an intention to quit within the next 30 days.^2^ Smoking cessation counseling can lead to sustained abstinence with the expected health benefits that come from such an effort.^3^ National organizations recommend the ED as a site for intervention.^4^ The importance of this topic is emphasized by a systematic review indicating that ED interventions, using a variety of methods, increases quit rates at 6–12 months from 3% in the usual care groups to 8–11% in the intervention groups.^5^

Given that smoking cessation counseling may be difficult to accomplish, a viable alternative to counseling in order to enhance a patient’s ability to quit smoking is through pharmacologic means.^6^ Nicotine replacement therapy from a variety of methods has been shown to increase the rate of successful cessation by 50%.^7^ One route of nicotine replacement therapy is with transdermal patches. The use of nicotine replacement therapy with patches (NRT-P) is associated with cessation rates of around 20% and rivals the success rate of varenicline or combination therapy.^8^

There is little data on the prescription fill rate for NRT-P for ED patients outside of a study environment. Not specific to NRT-P, authors have found 26% of ED patients failed to fill antibiotic prescriptions. Even when patients are prescribed opioid analgesics, more than 20% fail to fill their prescriptions.^10^ This finding highlights the rationale for the current study, as interventions with NRT-P prescriptions can only be successful if the patients actually obtain the medication. The purpose of this study was to evaluate ED prescription fill rates for NRT-P. We sought to determine factors associated with that fill rate. This information may help to guide further efforts to improve ED-initiated smoking cessation.

## METHODS

We conducted this study at an academic urban medical center ED and its three associated, freestanding community EDs in Northeast Ohio. The combined annual volume of these departments is approximately 150,000 patients. This was a retrospective chart review project conducted with institutional board review approval. Patients were identified through a search of our electronic health record (EHR) system (Epic Systems Corporation, Verona, WI). We identified all patients receiving an electronic prescription for NRT-P from January 2018–October 2019.

To evaluate fill rates, we leveraged the Surescripts functionality within the EHR, which allows evaluation of pharmacy benefit data from sources external to our health system. Surescripts collects and sends data from pharmacy benefits manager (PBM) transactions back into the EHR when queried. In addition, data from the health systems RX-30 ambulatory pharmacy system were imported back into the EHR through the Surescripts payer database to capture fill data from our internal pharmacies, regardless of patient payor status. Each time a patient has an encounter in the health system, including all subsequent ED encounters, outpatient clinic visits, refill encounters, and hospitalizations, the Surescripts system is queried, updating PBM data, including medication fills. Our EHR then stores the results of these queries in its Clarity database.

We compared NRT-P prescriptions from our EHR with the returned PBM and fill data. For the purposes of this analysis, we defined a “successful fill” as NRT-P dispensed from a pharmacy within one week of a NRT-P prescription from a provider in our system, a methodology that has been used by others to evaluate fill rates of other substance use disorder-related prescriptions.^11^ Since subsequent dispense queries are dependent on subsequent patient encounters, patients receiving a prescription for NRT-P who did not have a follow-up visit in our system as of the date of the query would not have data available. These patients were included in the initial dataset and then removed from subsequent analyses. Patients who received a paper prescription were not included in this database.

All charts were reviewed by one of the two study authors (CE and DT), both of whom are faculty members in the ED. A standardized data collection sheet was used to record the data. The review results of about 20 of the charts were done together between the two reviewers. Charts were reviewed to gather data on medical insurance, age, gender, purpose of ED visit, presence of chronic heart or lung problems, and evidence of other substance abuse. Chronic lung disease was evidenced by a history of asthma or chronic obstructive lung disease (COPD). Chronic heart disease was evidenced by a history of coronary artery disease, arrhythmias, or heart failure. Evidence of another substance use disorder was noted by a prior diagnosis in the chart of alcohol, cocaine, stimulant, opioid, or THC abuse. We reviewed subsequent clinic notes to determine whether patients had reported smoking cessation. If there was not specific evidence of smoking cessation then that patient was coded as continued smoking. No patients were excluded for any other reason aside from being a duplicate patient. We conducted statistical analyses to determine associations between fill rates and the factors noted above by chi-square analysis and Student’s t-test, as appropriate, using a *P* value of <0.05 to indicate statistical significance. The data is presented as the mean or percent with the 95% confidence interval (CI) in brackets.

**Population Health Research Capsule**

What do we already know about this issue?

*Nicotine replacement therapy is effective and recommended for implementation in the emergency department.*

What was the research question?

*Do patients fill their prescriptions for nicotine patch prescriptions?*

What was the major finding of the study?

*A total of 44% filled their prescriptions, with higher rates among women and those with chronic lung disease.*

How does this improve population health?

*Further research should focus on ways to improve the prescription fill rates and compliance with therapy to reduce smoking.*

## RESULTS

Our database search returned 598 unique patients prescribed NRT-P during the study period. We were able to determine NRT-P fulfillment rates on 500 of these patients with data from 68 unique pharmacies. The patients for whom we had follow-up data were similar to the patients without follow-up, with respect to gender, reason for ED visit (cardiac related, pulmonary related, or any other chief complaint), and history of substance abuse (including any substances other than tobacco) based on Student’s t-test or chi-square test, as appropriate. Those patients with follow-up data were older – 43.5 years [42.3–44.7] vs 39.7 [37.2–42.3], *P*<.05 – and more likely to have chronic lung disease 35% [31–39] vs 15% [12–18] p<.01, or cardiac problems 14% [11–17] vs 4% [2–6], *P*<.01, compared to those without follow up data. Patients who were self-pay were less likely to have return visits (24% [20–28]) compared with patients with any insurance (40% [31–51], *P*<.001).

The remaining 500 patients with follow-up data had an average age of 43.5 [42.3–44.7] years with 43.4% [39.0–47.9] being of female gender. The average time to fill for those patients for whom we had fill data was 0.7 days [0.6–0.8]. The average time for a follow-up visit, which is the time that the system would query the pharmacy was 116.9 days [107–127]. Sixty-two percent of patients were seen by an attending physician alone, while 17.6% were seen by advanced practice providers and 20.4% by resident physicians. Nineteen percent of patients presented with a pulmonary-related complaint, while 16% presented for chest pain or another cardiac-related complaint. Thirty-two percent of patients had a substance use disorder history. Overall, 13% of patients had evidence of smoking cessation documented in their chart. There was not a statistical association of prescription fill rate with cessation, although this study was not powered to assess this result.

Forty-four percent [39–48%] of patients filled their NRT-P prescriptions. We found no difference in the patients who filled their prescriptions based on age, prescriber type, reason for ED visit, presence of cardiac disease, or history of substance abuse disorder (Table 1). Patients of female gender and patients with a history of chronic lung disease were more likely to fill their prescriptions. With regard to medical insurance, self-pay patients were least likely to obtain their NRT-P prescriptions, (*P*<.01) (Table 2).

## DISCUSSION

We found that about half of patients prescribed NRT-P fulfilled their prescriptions. This is consistent with other studies of ED prescriptions that have found high rates of non-fulfillment for prescriptions of other medications.^10^,^11^ While studies in other environments have found higher fulfillment rates for NRT-P, they were in primary care practices or conducted in a study environment with greater resources available under study conditions. In prior studies of fulfillment of NRT-P outside of the ED, various authors have found non-fulfillment rates of around 20%.^12^,^13^ A review of available literature cited a number of methods of improving compliance with smoking cessation aids, many of which required more intensive intervention than is commonly available in the ED.^14^

We did not find a consistent relationship between a variety of factors and prescription fulfillment. There was not a significant difference based on age, history of substance use disorder, history of cardiac disorder, or purpose of the ED visit. Some studies have found such differences but with inconsistent results. A study among Medicare recipients, found that women were less likely to fill their prescriptions than men.^15^ A study of electronic prescriptions found higher non-redemption rates among men and younger men in particular.^16^ Studies of antidepressant medications have found that younger patients are less likely to fill their prescriptions.^17^

In our research, we found that patients with chronic lung diseases are more likely to fill their NRT-P prescriptions and more likely to quit smoking. The beneficial effects of a variety of interventions on smoking cessation have been verified in a meta-analysis of patients with COPD.^18^ Studies of asthmatic patients have found improved cessation rates with a variety of interventions including counseling and nicotine replacement therapy.^19^,^20^ For patients with COPD or asthma, intensive smoking intervention programs can lead to significant cessation rates.^21^ We did not find an association between cardiac disease and NRT-P fulfillment rates. Despite evidence of the efficacy of smoking cessation in patients with both cardiovascular disease and cerebrovascular disease, other studies have found low rates of cessation despite counseling.^22^ Studies have also found that intensive counseling programs, not generally available in the ED, may be effective in enhancing cessation.^23^ In line with the studies referenced above, further efforts to enhance prescription fulfillment along with counseling to support use of the NRT-P prescriptions would be expected to have beneficial health benefits.

## LIMITATIONS

We lacked pharmacy follow-up on 16% of our patients as they did not have a follow-up encounter in our system, which would have triggered a Surescripts query. We have no information on why patients may not have had a follow-up visit in our system and no information on patients who may have had subsequent encounters with area providers who do not use the Epic EHR system. In addition, since Surescripts only reports on PBM data from participating plans, patients in a non-participating plan or paying out of pocket (self-pay) at an external pharmacy would not have available data. We used a cutoff of one week for monitoring fulfillment of the prescription, consistent with other studies. A different cutoff might have been more appropriate for a chronic condition although then we might have had confounding from the effects of follow-up with other providers. We are dependent on the accuracy of the health record for information about comorbid conditions and insurance data. This methodology may not identify patients who obtained NRT-P by other means such as over-the-counter purchases or those who may have filled their prescription after their most recent follow-up encounter with our system. We are reliant on the accuracy of the health record for information about smoking cessation. Patients were not directly interviewed by the investigators.

## CONCLUSION

In our sample of ED patients, about half of patients prescribed NRT-P filled their prescriptions. Female patients, those with insurance, and those with chronic lung disease were more likely to fill their prescriptions.

## Figures and Tables

**Table 1:** Relationship with NRT-P* fulfillment.

Variable	Prescription filled N = 219	Prescription not filled N = 281	P-value
Age (years)	43.9+/− 12.7	43.2 +/−13.8	P=.92
Gender % female	52% [48–56]**	37% [33–44]	P<.01
Prescriber			P=.98
Attending	20%	20%	
Advanced practice provider	62%	63%	
Resident	18%	17%	
Reason for ED visit			P=.11
Pulmonary	21%	16%	
Cardiac	18%	15%	
Other	60%	69%	
Chronic lung disease % Yes	41%	31%	P<.05
Chronic cardiac disease % Yes	14%	13%	P=.75
Substance use disorder history % Yes	35%	32%	P=.41

* Nicotine replacement therapy-patches.

** Data is presented as the percent with the 95% confidence interval in brackets.

*ED*, emerency department.

**Table 2:** Relationship between insurance status and prescription fill.

Insurance type	Percent with prescription fill
Commercial	54% [41–66]*
Medicaid	62% [56–68]
Medicare	46% [33–58]
Self-pay	10% [4–20]

Difference between fill rates and insurance type P<.01.

* Data is presented as the percent with the 95% confidence interval in brackets.
